# Sporadic Meningioangiomatosis: A Series of Three Pediatric Cases

**DOI:** 10.7759/cureus.1640

**Published:** 2017-09-01

**Authors:** Raja Anand, Richard J Garling, Janet Poulik, Marko Sabolich, Dylan J Goodrich, Sandeep Sood, Carolyn A Harris, Abilash Haridas

**Affiliations:** 1 Neurosurgery, Wayne State University School of Medicine; 2 Pathology, Wayne State University School of Medicine

**Keywords:** meningioangiomatosis, intraoperative ultrasound, meningioma, nf2, neurofibromatosis

## Abstract

Meningioangiomatosis (MA) is a rare benign, hamartomatous lesion within the leptomeninges and cerebral cortex. Three percent of intractable epileptic patients with tumor develop MA. It may be accompanied with neurofibromatosis type II, or it may occur sporadically.

Three patients, age range of 2-16 years old, presented with episodes of seizure. The patients demonstrated no family history or stigmata of neurofibromatosis type II. Electroencephalogram (EEG) was unremarkable for epileptiform activity. Magnetic resonance imaging (MRI) revealed enhancing lesions within the frontal gyrus, the anterior cingulate gyrus, and the parietal lobe. Incomplete resection led to recurrence in one patient, and later, intraoperative ultrasound was used to achieve total resection in another patient. Each patient was seizure free on follow-up, and managed with anti-epileptic medication.

Resection is the only curative treatment in 85% of MA cases. Complete resection is necessary for symptomatic treatment in cases of MA, as recurrence has been documented in this lesion. Intraoperative ultrasound is an effective imaging modality to ensure gross total resection of MA.

## Introduction

Meningioangiomatosis (MA) is a benign, hamartomatous lesion typically described as an intracortical plaque-like proliferation of meningothelial or fibroblast-like cells around vessels [1-2]. First reported in 1915 [3-4], MA occurs in up to 3% of tumor patients presenting with intractable epilepsy [5]. MA may occur sporadically, or in association with neurofibromatosis type II (NF2) in up to 50% of patients [2, 6]. In patients with NF2, meningioangiomatosis often presents as an incidental finding on autopsy, since the majority of documented cases are asymptomatic. Sporadic cases of MA, however, are frequently accompanied by a history of seizures or headaches, with 81% of cases presenting with seizures as the chief complaint [7]. In this study, we present three cases of sporadic MA that presented to our center within the last three years.

## Case presentation

Case 1

**Figure 1:**
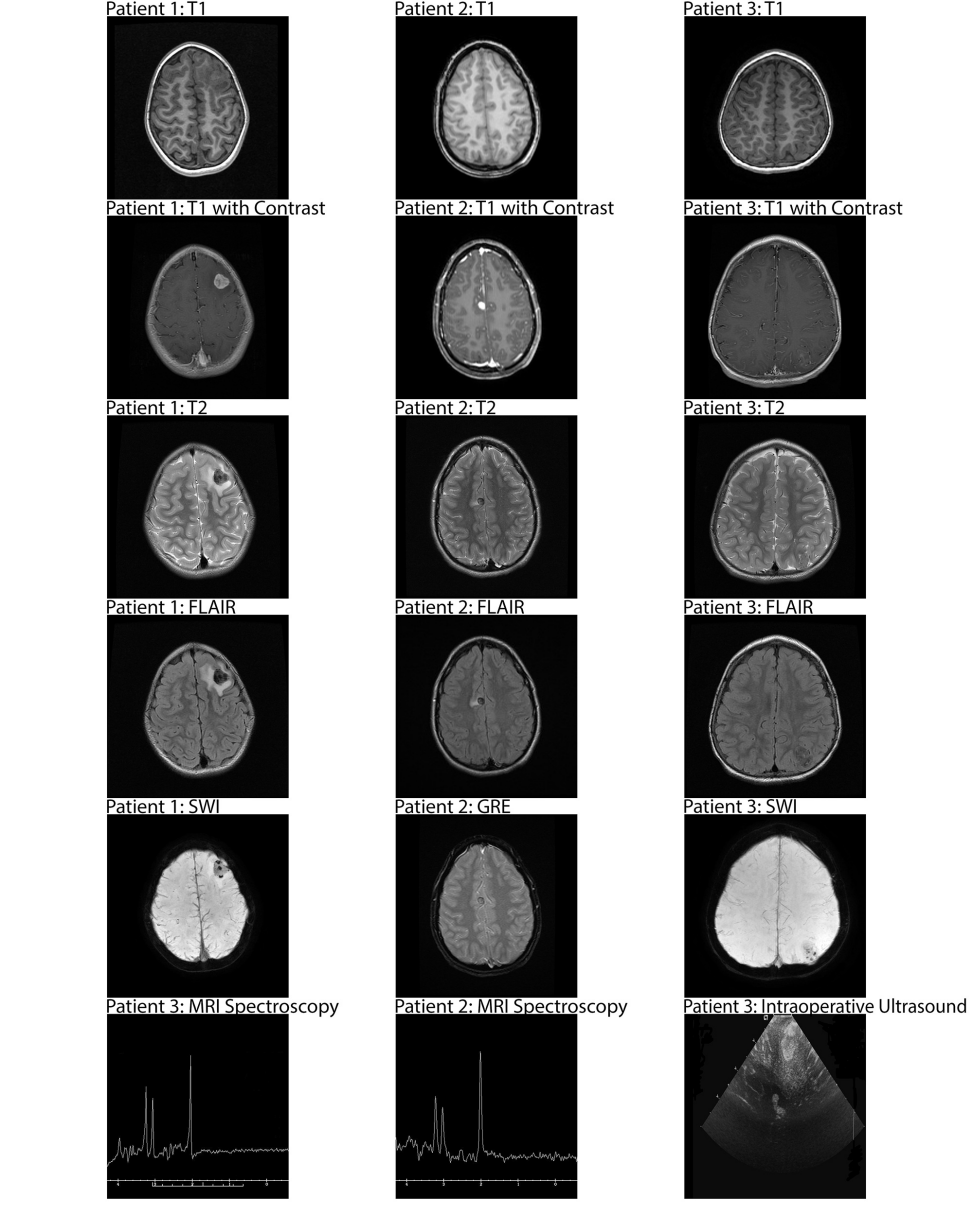
MRI brain, MRI spectroscopy, and intraoperative ultrasound of three patients treated in our center for MA. MA: Meningioangiomatosis; MRI: Magnetic resonance imaging.

The patient was a six-year-old girl with autism spectrum disorder and attention deficit hyperactivity disorder (ADHD) presenting with a one-month history of blank stares with whole body stiffening and rolling of the eyes. These events were reported to have occurred up to six times per week, routinely following bouts of anger or rage. Electroencephalogram (EEG) testing, however, was negative for seizures or any epileptiform activity (Table 1). Pre-operative magnetic resonance imaging (MRI) without contrast demonstrated an intraaxial, T1 isointense, T2 hypointense, intensely enhancing subcortical mass in the left medial frontal gyrus. Susceptibility weighted imaging (SWI) also showed significant susceptibility, indicating calcification. MRI spectroscopy yielded a mild elevation of the choline peak (Figure 1). No remarkable arterial enhancement was noted on magnetic resonance angiogram (MRA). No symptoms or family history of neurofibromatosis type II were found.

A firm, white, well-circumscribed mass was resected. Post-operative MRI brain fluid-attenuated inversion recovery (FLAIR) sequence demonstrated edema identical to pre-operative imaging. On three-month follow-up, the patient had a recurrence of seizures. Follow-up brain MRI seven months after resection revealed a dural-based enhancing lesion within the left frontal lobe that spread within the underlying site of surgery. Intraaxial and sharp dissection were performed, and a red, firm mass was excised. Post-operative brain MRI following the second resection again demonstrated linear enhancement along the anterior medial portion of the resection site, likely a post-operative dural reaction. SWI demonstrated significant susceptibility within the resection cavity, possibly due to blood product. The patient started levetiracetam to control seizure. Follow-up brain MRI 16 months after surgery showed no evidence of tumor recurrence. The patient was seizure free at 21-month follow-up and maintained prophylactically on oxcarbazepine (Table 1). The brain MRI acquired at 25 months after surgery showed no sign of recurring tumor.

Case 2

The patient was a 16-year-old girl suffering from intention tremors for several years. The tremors worsened one month prior to admission coinciding with episodes of gait instability, and nocturnal hallucinations (Table 1). The patient had also been suffering from headaches for two months, and emesis for six months. Pre-op MRI brain (Figure 1) revealed a T1 mildly hyperintense, T2 markedly hypointense, enhancing lesion superior to the right anterior cingulate gyrus. MRA findings were consistent with calcification or hemorrhagic etiology. The patient was prescribed levetiracetam for management of her seizures, and primidone for her tremors. No stigmata or family history of neurofibromatosis type II were evident. Internal debulking followed by circumferential resection was performed. The mass was white and very firm. No evidence of the lesion was found on post-operative MRI. Continued on levetiracetam for prophylaxis, the patient was seizure- and headache-free at two-week follow-up. MRI taken 19 months after surgery was negative for recurrence.

Case 3

The patient was a two-year-old albino male presenting with complex, tonic clonic seizures (Table 1). Fewer than five prior episodes of seizure were reported by the patient’s family. Episodes were 1-5 minutes in duration. Brain MRI revealed a left parietal enhancing lesion extending into the underlying white matter. The lesion was isointense on T1 weighted MRI and heterogeneous on T2 weighted MRI. SWI demonstrated punctate areas of susceptibility, suggestive of calcification. The lesion showed heterogeneous enhancement on MRI brain with contrast (Figure 1). The patient was administered levetiracetam for management of his seizures prior to surgery. No symptoms or family history of neurofibromatosis type II were found. Intraoperative ultrasound showed the left parietal lesion to be located within one major gyrus. Radical resection of the mass was performed. Ultrasound was again used to confirm total resection. Post-operative brain MRI was negative for residual tumor, and the patient was continued on levetiracetam. MRI five months after surgery was negative for enhancement within the resection cavity. The patient has remained seizure free.

**Table 1:** Three MA patients treated in our center. ADHD: Attention deficit hyperactivity disorder; EEG: Electroencephalogram; MA: Meningioangiomatosis; MRI: Magnetic resonance imaging.

Gender	Age	Chronic conditions	Presentation	EEG	Lesion location	MRI findings	Revisions	Antiepileptic drugs	Follow up
F	6	Autism Spectrum Disorder, ADHD	Blank stare episodes including whole body stiffening. No loss of consciousness	No epileptiform activity	Left medial frontal gyrus	T1 isointense, T2 hypointense, intensely enhancing lesion	One revision seven months after initial resection	Oxcarbazepine	Seizure free
F	16	None	Intention tremors in hands, visual and auditory hallucinations, and balance impairment episodes	No epileptiform activity	Superior to right anterior cingulate gyrus	T1 mildly hyperintense, T2 hypointense, enhancing lesion	No revisions	Levetiracetam	Seizure free
M	2	Albinism	Whole body tonic clonic, complex febrile seizures	No epileptiform activity	Right parietal gray matter mass extending into subcortical white matter	T1 isointense, T2 heterogeneous hypointense, enhancing lesion	No revisions	Levetiracetam	Seizure free

Pathology

Case 1

**Figure 2:**
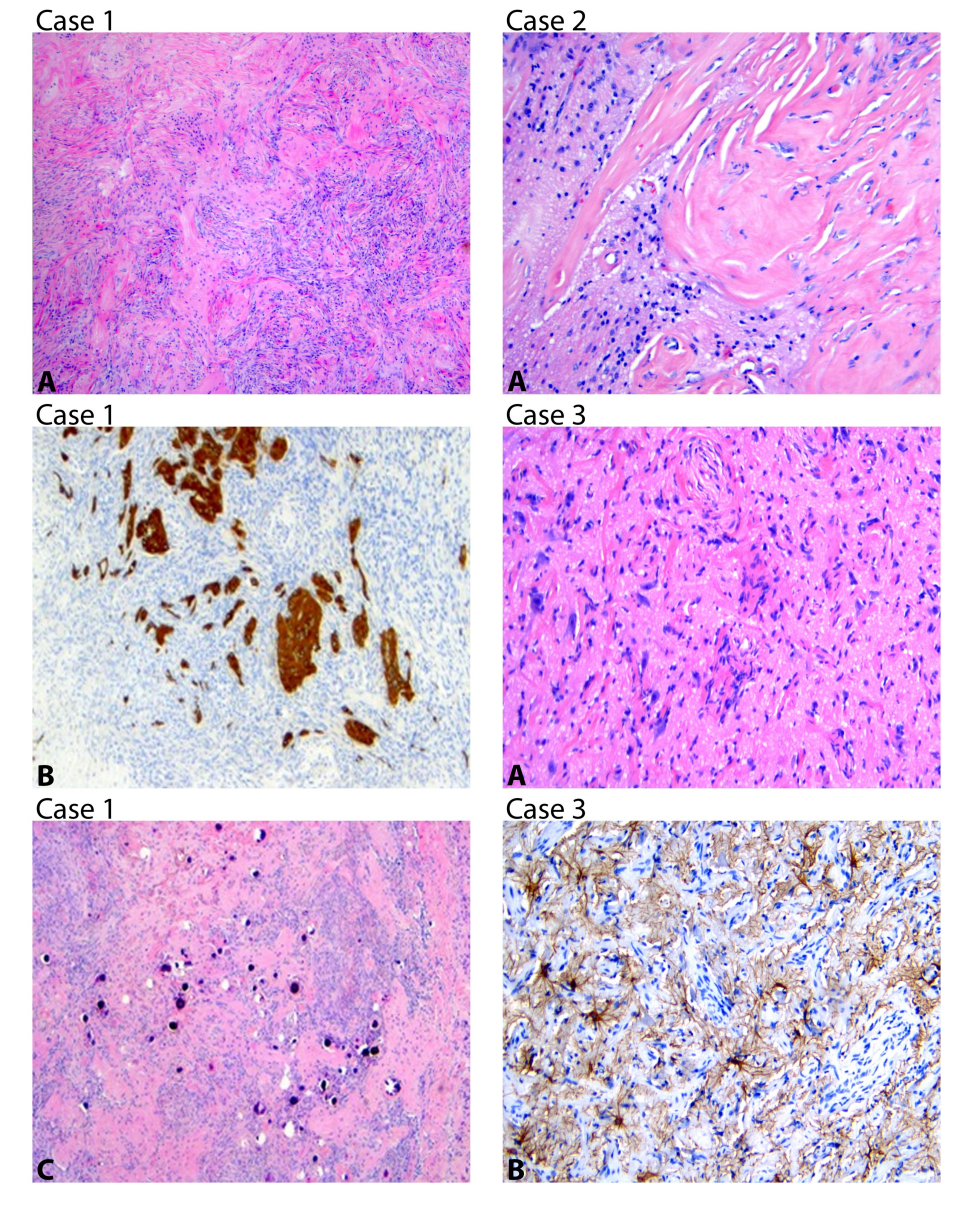
Case 1: (A) Meningothelial proliferation infiltrating into brain parenchyma H&E (4X). (B) GFAP highlighting entrapped gliotic brain parenchyma (H&E 10 X). (C) Numerous psammomatous calcifications H&E (4X). Case 2: (A) Irregular interface between dense fibrous tissue and brain parenchyma H&E (10X). Case 3: (A) Small caliber vessels and meningothelial perivascular meningothelial fibroblast-like proliferation illustrating invasion into brain parenchyma with entrapped and slightly enlarged neurons H&E (10X). (B) GFAP immunoreactivity in astrocytes (10X).

Histologic assessments (Figure 2) showed a fibrous proliferation consisting of meningothelial cells and dense collagen infiltrating into cerebral cortex and entrapping gliotic brain parenchyma confirmed by a GFAP immunostain. Areas of hypocellular fibrocollagenous tissue with whorls and numerous psammomatous calcifications were noted. The Ki-67 index was 3-5%, indicating mild proliferation. Small foci of intraparenchymal hemorrhage were evident within the overlying cortical parenchyma, however, no vascular malformations were seen.

Case 2

Pathological samples (Figure 2) displayed dense fibrous connective tissue interfacing with brain tissue. At the interface, the connective tissue formed a network of sclerotic vessels of varying density entrapping both gliotic cortex and white matter. The larger mass’s connective tissue exhibited a lobular arrangement and included small cellular foci composed of irregular spindle cells, small mononuclear inflammatory cells, and scarce calcifications, including psammoma bodies. CD34 highlighted vascular channels, including lumens of many sclerotic vessels. Ki-67 highlighted a few cells, mainly within the cellular foci.

Case 3

Pathological analysis (Figure 2) exhibited a plaque-like lesion with meningothelial and vascular proliferation. Focal areas of calcification were evident. Numerous psammoma bodies were identified. CD34 highlighted an increased number of small vessels, while GFAP, S100, and Neu-N highlighted scattered neurons.

## Discussion

Meningioangiomatosis is a rare tumor, with fewer than 100 published papers to date. Its precise pathophysiology is still unknown. Theories of dysplasia or reactive etiology exist due to MA’s benign nature and lack of proliferation in most cases [2, 7]. Its Ki-67 index has been reported as low as 0.1% [3]. The majority of MA lesions present as solitary, well-demarcated masses within the leptomeninges and underlying cortex. The differential diagnosis includes oligodendroglioma, granulomatous meningitis, meningioma, parasitic disease, and calcified vascular malformation [2]. Rarely, MA has occurred concurrent with cerebral meningioma. Additionally, cystic MA has been documented in the literature [3]. The majority of cases, however, are sporadic, with no family history or stigmata of neurofibromatosis type II. These patients typically present with refractory seizures and/or episodes of headache. A literature review conducted by Jallo, et al. in 2005 found that sporadic MA has a mean age of 18.6 years, and is mostly in the temporal and frontal lobes [2, 5-6]. The operative course is typically unremarkable for recurrence, except in cases of incomplete resection. Surgery is necessary for seizure and headache relief in 85% of sporadic cases, with some patients maintained on anti-epileptic drugs (AEDs) for further prevention of epileptic recurrence [7]. Gross total resection is necessary in most cases as even use of several anti-epileptic drugs may not be effective for intractable seizure control [1].

Each patient was studied via 12-hour or 24-hour EEG to assess epileptic discharge. Notably, all three patients were negative for both interictal and episodic anomalies on EEG. Therefore, MA may be considered in cases of non-epileptic seizure.

MA is most often hypointense on T1 weighted MRI and hyperintense on T2 weighted MRI. Furthermore, 80% of masses in MA will enhance on T1 brain MRI. Additionally, 89% of MA cases in the literature show calcification on computed tomography (CT) scan [6]. All three cases in our study enhanced on MRI (Table 1), with extensive calcification on SWI. Therefore, MA should be considered in the differential for cases presenting with calcification and meningothelial invasion.

Only one case to our knowledge has been reported regarding the use of ultrasound to image meningioangiomatosis in humans [8]. Ultrasound is useful as an intraoperative tool that ensures gross total resection is achieved in cases of extensive meningothelial proliferation. Recurrence in our first case was due to incomplete resection secondary to infiltration of the surrounding dura. Although the lesion was excised circumferentially, remnants of the tumor proliferated and seizures returned three months after surgery. Intraoperative ultrasound, however, was used successfully in the third patient to provide complete and total resection of the tumor (Figure 1). On follow-up, all three patients were maintained on AEDs and were seizure free.

The cases were notable in their chronological proximity and patient ages. The three cases presented within a two-year period, suggestive of clinical relevance within the pediatric population. As noted, the mean age of presentation for sporadic MA is 18.6 years. However, patients ranged from a second-trimester fetus to a 73-year-old female in our review of the literature [8-9]. Therefore, MA may be considered in cases of refractory seizures in all age groups. MA’s prevalence in all age groups may be attributed to its ability to silently proliferate for years prior to symptoms of epilepsy, or for life if associated with NF2.

## Conclusions

Meningioangiomatosis, although benign, is a causative factor for symptoms of epilepsy. Surgical intervention can reduce or eliminate seizures. Intraoperative ultrasound appears to be an effective tool to ensure complete resection.
